# A virtual evaluation of options for managing risk of hospital congestion with minimum intervention

**DOI:** 10.1038/s41598-022-18570-5

**Published:** 2022-08-27

**Authors:** Wanxin Hou, Shaowen Qin, Campbell Henry Thompson

**Affiliations:** 1 School of Information Science and Technology, Research Centre for Intelligent Information Technology, Nantong University, Nantong, Jiangsu China; 2 College of Science and Engineering, Flinders University, Adelaide, Australia; 3 School of Medicine, University of Adelaide, Adelaide, Australia

**Keywords:** Health care, Risk factors

## Abstract

Hospital congestion is a common problem for the healthcare sector. However, existing approaches including hospital resource optimization and process improvement might lead to huge cost of human and physical structure changes. This study evaluated less disruptive interventions based on a hospital simulation model and offer objective reasoning to support hospital management decisions. This study tested a congestion prevention method that estimates hospital congestion risk level (R), and activates minimum intervention when R is above certain threshold, using a virtual hospital created by simulation modelling. The results indicated that applying a less disruptive intervention is often enough, and more cost effective, to reduce the risk level of hospital congestion. Moreover, the virtual implementation approach enabled testing of the method at a more detailed level, thereby revealed interesting findings difficult to achieve theoretically, such as discharging extra two medical inpatients, rather than surgical inpatients, a day earlier on days when R is above the threshold, would bring more benefits in terms of congestion reduction for the hospital.

## Introduction

Hospital congestion is becoming a major concern in this modern era due to increasing patient demands. More hospital congestion episodes accompanied by longer waiting times and queuing length have been associated with a greater risk of hospital-acquired infections, public complaints and, possibly, negative impacts on hospital staff mental health^1,2^. Hence, there is an urgent need to find effective ways to reduce overcrowding and congestion in hospitals.

Recent studies have sought solutions from various perspectives and with various approaches (Table 1). A considerable amount of attention has been directed to explore solutions for crowding within some parts of the hospital, particularly the Emergency Department (ED), aiming to reduce access block through improving ED performance^3–9,11–13^. However, to deal with more general, hospital-wide problems, instead of focusing on a particular department, examining the hospital as a connected whole based on real data is necessary. This is because the complicated interactions among these parts must be taken into consideration^14^.

**Table 1:** A summary and comparative review of the major works in the literature.

Category	References	Tools	Objectives	Study areas
Resource adjustment	Brenner et al.^3^	Simulation	Finding an optimal number of resources	ED
	Zeinali et al.^4^	Simulation-based metamodeling	Resources planning	ED
	Ghanes et al.^5^	Simulation	Optimizing human resource staffing level	ED
	Chen et al.^6^	Multi-objective simulation optimization	Resource optimization to reduce hospital congestion	ED
	Hajjarsaraei et al.^7^	Simulation	Human resource planning	ED
	Diefenbach et al.^8^	Simulation	Analyzing the effect of bed configuration	ED
	Paul et al.^9^	Simulation	Optimizing bed utilization including additional resources	ED
	Hejazi^10^	Simulation	Resource optimization and planning	Whole hospital
Process improvement	Kaushal et al.^11^	Simulation	Evaluation of fast track (additional non-urgent areas) to reduce congestion	ED
	Hussein et al.^12^	Simulation /Six Sigma	Changing utilization technology of medical equipment or introducing new equipment	ED
	Liu et al.^13^	Simulation-based optimization	Improving the efficiency of ED unit	ED

ED, emergency department.

In terms of methodology, there have been many studies attempting to reduce hospital^3–13^ congestion and improve the efficiency of hospital operation using simulation-based approach. This approach allows researchers to digitally represent and capture the variability, uncertainty, and complexity of a dynamic system^14^. Compared to analytical methods, simulation-based approach is more suitable for examining system-wide consequences of changes in one or more areas of the hospital in a risk-free environment^4^.

In terms of hospital improvement strategies, many studies focused on optimizing human and physical resources utilization or using additional resources in order to improve hospital performance^3–11^. Other studies introduced process improvement strategies to address hospital overcrowding issues. For example, a so-called fast track strategy that includes discharging low-acuity patient quickly while improving the schedule of patients’ discharge and establishing a fast-track service line was proposed to accelerate hospital process^15^.

Definitely, resource optimization and process improvement are resultful ways to reduce hospital congestion. However, from a feasibility viewpoint, it is potentially difficult to implement these approaches because they tend to involve large human and physical structure changes. Naturally, achieving better results through applying a less disruptive intervention (smaller adjustments leading to satisfactory outcomes) would be managerially more desirable. To our knowledge, research on identifying such less disruptive interventions is still lacking. This study hopes to fill this gap in the literature with more feasible interventions to control hospital crowding.

This paper introduces a congestion prevention method that aims to manage the risk of hospital congestion with less disruptive interventions. Through virtual implementation of this method using a simulation model, this study demonstrates, with highly detailed simulation results, the effectiveness of both the prevention method and the virtual implementation approach. In the rest of this paper, “Methods” section mainly introduces the congestion prevention method and simulation-based evaluation. “Results” section exhibits the simulation results so that the solutions can be compared and considered for real-world disposition. Discussions and conclusions are presented in “Discussion” and “Conclusion” sections.

## Methods

### A hospital congestion prevention method

This section introduces a research-based congestion prevention method based on a concept named “hospital’s instability wedges”^16^. The concept demonstrates that, theoretically, the risk of patient flow congestion can be calculated on a daily basis and prevention can be achieved by activating interventions that involve a very small number of patients when the risk level is deemed high. This leads to a potentially effective method for real-time congestion prevention.

The typical scenario in a hospital is that, on a daily basis, patients arrive at the ED via ambulance or self-presentation. Some patients are discharged from the ED and some admitted. Apart from ED, inpatients are discharged after a period of stay, and a variable number of planned elective admissions occur. These processes compete for limited resources and congestion episodes occur when bottlenecks appear. The method first estimates the risk of congestion *R* at a set time every day, using hospital occupancy, predicted patients’ admission and discharge numbers based on the day-by-day variation patterns derived from the hospital’s history data, and activates minimum intervention when the risk *R* is above a certain level. The risk *R(C)* represents the ratio that the current day’s midnight occupancy (*M*_*t*_) exceeds a specified threshold *C*. The threshold *C* was set as the hospital’s normal bed capacity in this study. The probability of such exceedance *R(C)* was calculated by the following equation:

1 $$R(C) \approx P(M_{t} > C)$$

In order to control the risk of hospital congestion, this formulation has been refined to become more implementable and controllable for decision-makers. The following equation was considered at the beginning:

2 $$M_{t} = \eta_{t} N_{t} + E_{t} (1 - \omega_{t} ) + M_{t - 1} (1 - v_{t} )$$

where *N*_*t*_ indicates the new arrivals at the hospital ED on the day. *E*_*t*_ is defined as the elective patients scheduled to stay in the hospital at least one night. *M*_*t*−1_ is the midnight occupancy of the previous day. $\eta_{t} \in (0, 1], \omega_{t} \in (0, 1],v_{t} \in (0, 1]$ denotes the admission rate of the new arrivals, cancelled elective patients and inpatients discharged, respectively.

The following task is to investigate the dependence of the congestion risk *R*(*C*) on $\eta_{t} ,{ }\omega_{t} {\text{and}} v_{t}$ which are treated as control parameters. Due to the fact that *N*_*t*_ is the only random variable on the right-hand side of Eq. (2), Eq. (1) can be refined as follows:

3 $$\begin{aligned} R(C) & = P(M_{t} > C) \\ & = P\left( {N_{t} > [C - M_{t - 1} (1 - \nu_{t} ) - E_{t} (1 - \omega_{t} )]\frac{1}{{\eta_{t} }} } \right) \\ \end{aligned}$$

**Figure 1:**
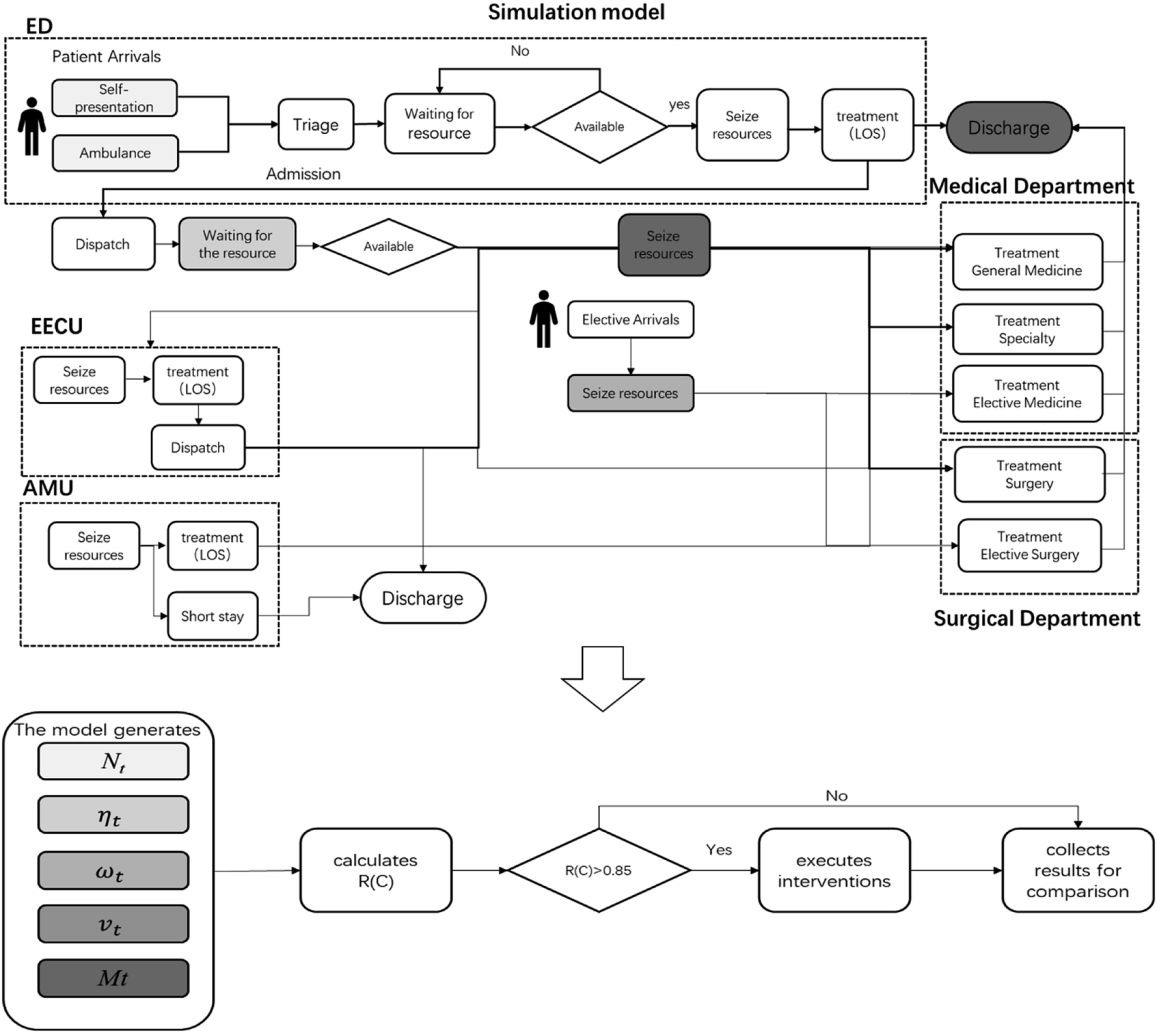
Process of the simulation-based evaluation.

As a result, *R(C)* became the simplified notation $\it {\text{R}}\left( {{\text{C}},{ }\eta_{t} ,\omega_{t} ,v_{t} } \right)$ which is composed of the control parameters. *Nt* fits the normal distribution (See Histogram of *Nt* in Supporting Information SI-Fig. 1).

Equation (3) can be refined again based on the simple restriction $\omega_{t} = 0$:

4 $$R(C) = r(C,\eta_{t} { },0,\nu_{{t{ }}} ) = P(N_{t} > x_{t} ) = 1 - {\Phi }\left( {x_{t} ;\mu_{t} ,\sigma_{t}^{2} } \right)$$

where $x_{t} : = [C - M_{t - 1} (1 - \nu_{t} ) - E_{t} ]{/}\eta_{t} {,}\;\mu_{t}$ and $\sigma_{t}^{2}$ are the mean and variance of the population of arrivals in ED on the selected day of the week and $\Phi {(}x;\mu ,\sigma^{2} {)}$ denotes the probability distribution function of the normally distributed random variable $X \sim N(\mu ,\;\sigma^{2} )$. Consequently, *R(C)* can be easily calculated based on the equation above.

This method allows decision-makers to change the control parameters to prevent hospital congestion when the congestion risk *R(C)* is high. That is to say, if we adjust the threshold *C* or the admission rate $\eta_{t}$ or discharge rate of patients $v_{t}$, *R(C) *will be changed accordingly. The method can be applied to different hospitals. In this study, data from a large Australia metropolitan hospital was collected for testing the method. An example of *R(C)* change with different $\eta_{t}$ and $v_{t}$ of the hospital was showed in SI-Fig. 2. It demonstrated that smaller changes in the numbers of admitted or discharged patients exhibit more effective and sensitive impacts on *R(C)*.

We assumed that hospital managers check the congestion risk using this method in the morning and adjust the plan for the day. If the risk rate is extraordinarily high in the morning, the probability of congestion occurrence might also be high during the day. Therefore, managers will plan to make an adjustment to the bed capacity or discharge a few patients to weaken this discordant possibility. Relying on the above method using historical patient flow data allows us to understand the change of congestion probability *R(C)* when interventions are adopted. However, it is impossible to demonstrate the expected, but more intuitive and quantitative, impacts on congestion episodes only by the method. Moreover, when managers attempt to manipulate different numbers of patients for congestion prevention, other issues might emerge including the type of patients or department that should be the focus for the intervention(s). In other words, from a managerial perspective, the type of patients affected by an intervention can impact on de-congestion effectiveness. Smaller adjustments affecting several different types of patients may be more effective and sensitive in reducing congestion risk. To address these issues, simulation modelling carries an advantage due to the fact that it provides a risk-free platform to help stakeholders access changes in operations, managerial policies and examine different alternatives. Through implementing the method and designing more specific indicators for hospital congestion based on the simulation model, the impacts of less disruptive interventions on congestion prevention were investigated explicitly and in depth.

### Simulation-based evaluation

Aiming to provide more accessible service is not a simple task for the healthcare system because hospitals are complex and dynamic. To achieve hospital service improvement, a comprehensive modeling of the complex system named Hospital Event Simulation Model: Arrivals to Discharge (HESMAD) has been developed by the tool named Anylogic in order to imitate the dynamic behaviours necessary for, and consequent to, each theoretical intervention^17^. The structure of HESAMD was showed in SI-Fig. 3. The model was constructed to simulate behaviours of the hospital as realistically as possible due to connecting to 2 years’ real patient flow data (2014–2016) of the large Australia metropolitan hospital. Definitely, it can be applied to different hospitals if patient flow data are provided. It contains several components representing emergency admissions, elective admissions, inpatients and discharge. The whole process and modules of HESMAD were described in SI4.

In order to clarify the decongestion effects of different strategies more thoroughly, the less disruptive intervention ideas from the method were transcribed into scenarios for investigating in the simulation model (Fig. 1).

We assumed that hospital managers check the congestion risk using the method in the morning and adjust the plan for the day. When *R(C)* exceeded 0.85, managers could add beds or cancel operations on a few patients for that particular day. In the simulation platform, the same process was realized. The model calculates *R(C)* in 8:00 am every day. If *R(C)* > 0.85, the intervention is executed for that day. All parameters used for *R(C)* calculation are generated by the simulation model on a daily basis. Furthermore, a color-coding system was also adopted for each scenario evaluation before, during and after hospital overcrowding.

The threshold was defined as the hospital capacity in the risk prevention method in this study. In this large tertiary hospital, there are 330 base beds including 170 medical beds, 130 surgical beds and 30 AMU beds in separate inpatient departments. 8 flexible beds can be arranged when the hospital is nearing exhaustion of its finite capacity. Using the congestion risk prediction method, the impacts of adding flexible beds on congestion prevention (scenario 1–4) were estimated by the HESMAD model. In order to evaluate the effects of less disruptive interventions, smaller adjustments were always promoted at the beginning. Furthermore, the department to which the flexible beds are added (scenario 5, 6) can influence decongestion efficiency was investigated (Table 2).

**Table 2:** Threshold and discharge scenarios.

Scenario No	Threshold	Description
**Threshold scenarios**		
0	330	Base case (No flex beds added)
1	332	2 flexible beds added (1 bed for medical 1 bed for surgical department)
2	334	4 flexible beds added (2 beds for medical 2 beds for surgical department)
3	336	6 flexible beds added (3 beds for medical 3 beds for surgical department)
4	338	8 flexible beds added (4 beds for medical 4 beds for surgical department)
5	338	8 flex beds for medical department
6	338	8 flex beds for surgical department
**Discharge scenarios**		
7–10	330	Discharging 2, 4, 6, 8 inpatients
11–14	330	Discharging 2,4,6,8 medical patients
15–18	330	Discharging2, 4, 6, 8 surgical patients
19–20	330	Discharging 2, 4 long stay patients (LOS > 21 days)
**Admission scenarios**		
21–24	330	Remove 2, 4, 6, 8 patients planned to be admitted

In the simulation platform, the discharging intervention was transcribed into different scenarios to test its effects on hospital congestion. However, the type of patients which is more likely to impact on congestion could be an issue. Discharging operations without considering patient types from the method is not sufficient. Therefore, interventions on different types of patients were executed by the simulation model on a daily basis but only when the congestion risk rate reaches or exceeds 0.85 (scenario 10–21) at 8 am each day (Table 2). In practice, low-acuity patients (e.g., patients with fracture or with chronic disease) are always selected for early discharge. Also, from an ethical aspect, those patients who have recently started treatment are not considered for discharge. In order to realize the patients’ selection for discharge by the simulation model, the model selected patients who have 1 day left of their hospital stay and whose triage score ≥ 4 for discharging (SI--Tables 1, 2: Triage). The simulation model generates patients who were assigned all information including Length of stay, triage number and personal information related to the whole treatment process, therefore, discharging low-acuity patients is easily realized. Since this study concentrates on the congestion prevention of differing inpatient departments, the model was adjusted for inpatients including medical, surgical and long stay patients (SI-Table 2).

Controlling admission rate was also tested by the simulation model for congestion prevention. In ED, 2–8 patients who are planned to be admitted were removed when *R(C)* reaches 0.85.

A color-coding system, similar to traffic signals used to trace hospital overcrowding status, was adopted into the HESMAD on a daily basis.

- Green day means that the hospital has at least 10% of total inpatient beds available.
- Amber day means that the hospital has between 3 and 10% of total inpatient beds available.
- Red day means that the hospital has less than 3% of total inpatient beds available.

The accumulated numbers of green, amber and red days were collected finally to indicate the congestion situation. Also, the midnight hospital occupancy, R(C), and the number of patients affected by each intervention were recorded every day for each simulation-based evaluation. Each simulation runs for 2 years. Results of the second year were collected for analysis to minimize the effect of the ‘warm-up’ period of the first year. In addition, each scenario was replicated 20 times under the same condition to obtain an average behaviour that would allow meaningful comparison of the results from different intervention scenarios. Minimizing the number of red days was the goal for different interventions. The reduction in the number of red days per affected patient was also calculated to estimate the efficiency of each scenario whereby the efficacy of each intervention is related to its disruption to patient care. The result comparisons are exhibited in Table 3.

**Table 3:** The results of scenarios (20 replications).

Scenario No	Midnight occupancy	Mean standard deviation	Numbers of red days	Numbers of amber days	Numbers of green days	Patients affected	Red days reduction per affected patient
0	311.8	4.16	79.95	250.6	33.45	–	–
1	308.21	4.53	53.1	232.2	79.7	–	–
2	310.22	4.71	45.45	240	79.55	–	–
3	310.75	4.63	44.6	229.9	90.5	–	–
4	311.26	4.33	37.2	220.45	107.35	–	–
5	308.23	4.75	23.24	204.95	136.81	–	–
6	313.51	4.23	50.25	231.1	83.65	–	–
7	310.38	4.64	67.45	252.75	43.8	225.6	0.055
8	310.18	4.32	63.4	255.35	45.25	446.4	0.037
9	309.87	4.88	60.7	256.15	47.15	597.6	0.032
10	308.57	4.23	54.75	260.8	48.45	816	0.031
11	306.64	4.41	52.21	234.42	77.37	278.4	0.100
12	305.77	4.32	50.95	227.6	85.45	417.6	0.069
13	304.85	4.46	43.5	226.25	94.25	518.4	0.070
14	304.45	4.86	41.05	229.2	93.75	681.6	0.057
15	311.04	4.63	70.65	254.85	38.5	235.2	0.040
16	310.23	4.56	68.75	249.95	45.3	465.6	0.024
17	309.84	4.37	66.3	249.2	48.5	691.2	0.020
18	309.41	4.23	63.15	246.65	54.2	844.8	0.020
19	310.63	4.53	70.4	252.45	41.15	134.4	0.071
20	310.19	4.12	64.1	254.45	45.45	247.3	0.064
21	309.45	4.52	62.56	254.17	47.27	220.3	0.078
22	309.12	4.41	61.31	256.31	46.38	450.2	0.042
23	308.23	4.33	58.64	260.42	44.94	590.5	0.036
24	307.64	4.15	53.29	266.74	43.97	805.3	0.033

Access to anonymised patient flow data used in this study was granted through an ethics approval process governed by Research Governance & Ethics-Office for Research, Southern Adelaide Local Health Network who waived informed consent of participants. We confirm that all methods were carried out in accordance with relevant guidelines and regulations.

## Results

The base case scenario in Table 3 is the baseline for the result comparisons of different interventions. The results of scenario 1–6 indicated that the occupancy increased slightly when the total bed capacity increased. The number of red days decreased from 79.95 to 53.1 when 2 flexible beds were added to inpatient departments (scenario 1) compared to base case scenario. The more flexible beds were added, the greater the reduction in red days. By contrast, the number of green days changed from 33.45 to 107.35 days when elevated threshold interventions were executed (scenario 1–4). The number of amber days decreased accordingly while implementing scenario 1–4. This was a non-linear decrement because the number of amber days of scenario 3 was 240 which increased slightly compared to scenario 2. Adding 8 flexible beds to the medical department (scenario 5) resulted in a 70.93% reduction of red-days comparing with the base-case scenario (scenario 0). However, adding 8 beds to the surgical ward (scenario 6) only achieved a 37.15% reduction in red days.

Scenario 7–10 focused on earlier discharges of different numbers of inpatients. According to Table 3, the midnight occupancy decreased from 311.8 to 308.57 while discharging 2–8 inpatients. Discharging 2–8 inpatients (scenario 7) when *R(C)* exceeds 0.85 resulted in 15.63%, 20.7%, 24.8% and 31.52% decreases in red days respectively compared to base-case scenario (scenario 0). Differing from threshold scenarios, the amber days increased from 250.6 to 260.8 when 2–8 inpatients were discharged (scenario 7–10). These scenarios also offered increases of 31%, 35%, 41% and 45% in green days respectively. Compared to discharging inpatients, controlling admission rates seems to achieve more red days reductions. Removing 2–8 inpatients (scenario 21–24) led to 21.75%, 23.31%, 26.65% and 33.35% decreases in red days.

The midnight occupancy decreased from 311.8 to 304.45 while executing scenarios 11–14. Discharging medical patients produced greater levels of red-days reduction compared to other discharging scenarios. Removing 2 medical patients when *R(C)* exceeds 0.85 led to a 34.7% reduction in red days (scenario 1). Particularly discharging 8 medical patients generated a 48.66% red day reduction. Amber days increased 6.46%, 9.18%, 9.72% and 8.54% respectively when 2–8 medical patients were discharged. Discharging medical patients also achieved green days increase from 33.45 to 97.

For surgical patients, these discharging interventions maximally reduced red days by 21.01% (scenario 18). Removing 2–8 surgical patients (scenario 15–18) can boost the number of green-days from 38.5 to 54.2, but these particular interventions had only a limited impact on the number of amber days. The midnight occupancy was decreased from 311.8 to 309.41.

Removing 2 and 4 long stay patients when* R(C)* exceeds 0.85 resulted in 11.94% and 19.82% reductions of red days. The number of amber days slightly increased. Also, the number of green days increased 23% and 36% respectively when 2 and 4 patients were discharged. These two scenarios only have limited impacts on the change of midnight occupancy (311.8–310.19).

Red days reduction per affected patient was also calculated to evaluate the efficiency of each scenario in Table 3. Red days reduction per affected patient of discharging two medical patients was 0.1 which was higher than discharging two surgical (0.04) or two long-stay patients (0.07). This suggested that a discharge strategy is more effective and less disruptive if medical patients are discharged. The other discovery was that for all discharging scenarios, the efficiency of red days reduction of removing smaller numbers of patients is always greater than discharging more patients.

## Discussion

This study investigated the congestion prevention method in the simulation model to investigate the potential impacts of different approaches especially less disruptive interventions on hospital overcrowding. In this study, a colour-coding system which is similar to traffic signals was adopted to describe the status of hospital overcrowding and used it for results comparison of different scenarios. The results demonstrated that threshold scenarios were more effective for red day reductions than discharging scenarios. We considered that adding a bed might benefit a considerable number of patients during a period of hospital congestion. Discharging patients seems to have fewer effects because it only involves certain numbers of discharged patients. However, it is important to recognize that opening a bed requires more costs, discharging patients saves money. Hence, from a cost–benefit perspective, the profits and costs from decongestion effects of the scenarios need to be explored further in the future tasks.

Another interesting discovery is that adding 2 beds hugely decreased red days, however, adding 4 beds and 6 beds have very similar effects on red-days reductions. One possibility is that some patients waiting in the queue are admitted to these additional beds which sustains the occupancy, consequently, the red-days gap between adding 4 beds (scenario 2) and 6 beds (scenario 3) is not obvious. It demonstrated that a smaller change seems to be more efficient.

Adding beds especially in the medical department brings more expected benefits in respect of congestion reduction for the hospital. Also, discharging medical patients rather than surgical patients brings benefits in respect of congestion prevention and leads to impressive red-days reductions and elevations in the numbers of green-days. To seek to understand this phenomenon, by tracing the historical data (SI-Table 3), it has been found that there are 17.3% more medical patients than surgical patients. Also, the proportion of medical patients whose LOS exceeds 21 days is higher than that of surgical patients. The total period of time where hospital beds are occupied by medical patients for one year is longer than surgical patients. Consequently, we believe that medical patients contribute to hospital congestion more significantly than other types of patients. When interventions are implemented for medical patients and medical departments, the effect on red days reduction is more obvious.

In this study, the cumulated numbers of red days, amber days and green days for each one-year simulation period were recorded based on the colour-coding system. A reduction in the number of red days is the common goal of all interventions. However, for the change of amber days and green days, we still need to discuss further. In the face of a reduction in red days, there are three patterns of possible change for the number of amber and green days (Table 4). If, when red days decrease, an intervention can lead to amber days decreasing and green days increasing, this suggests decongestion is occurring. But this might be construed as inefficient in terms of a resourcing perspective. From the utilization efficiency point of view, the preferable operating pattern of the hospital is that resources are utilized as much as possible while patients can still flow smoothly. That is to say, a more desired consequence of decongestion or red day reduction is an increase in the numbers of both amber days and green days, such as pattern 2 in Table 4. It has been seen that the amber days increase, but the green days decrease in pattern 3 in Table 4. In this case, decision-makers should check parameters such as queue length of patients waiting for the treatment and midnight occupancy to confirm that patients still flow smoothly. Otherwise, this latter pattern of intervention has a limited effect on hospital overcrowding. Discharging inpatients (scenario 7–10) especially discharging smaller numbers of surgical patients (scenario 15) and long-stay patients (scenario 19) will slightly increase amber days which belongs to pattern 2 in Table 4. Hence, less interruptive discharging interventions are also preferred from the utilization efficiency perspective.

**Table 4:** Three patterns of outcomes.

	Red days	Amber days	Green days	Scenario No
Pattern 1	↓	↓	↑	1–6, 11–14, 16–18
Pattern 2	↓	↑	↑	7–10, 15, 19–20
Pattern 3	↓	↑	↓	21–24

We also calculated the ratio of the reduction in red days expressed relative to the number of patients affected by each intervention and we called this the efficiency of each scenario. We discovered that for different types of patients, red days reduction per affected patient of removing fewer patients is always more favorable than discharging more patients. This finding confirmed “the hospital instability wedge” phenomenon which demonstrated that a less disruptive intervention applied may be a more cost-effective way to address congestion risk.

In this study, the congestion prevention method was adopted to calculate *R(C) *which provides the condition to execute a range of interventions. The value of 0.85 was selected as the threshold for scenarios execution. Scenarios were also tested for different *R(C)* values (SI-Table 4). It demonstrated that selecting *R(C)* > 0.85 for the condition maximises occupancy and benefits for the least disruption to patient care.

It must be recognized that a large amount of effort was made in HESMAD validation^17^. However, it is important to keep in mind that the simulation study does not attempt to propose exact mechanisms for hospitals. Rather, the simulation results demonstrate where greater attention should be paid when addressing patient flow congestions within a hospital if improvements are desired.

## Conclusion

This study investigated the congestion prevention method in the simulation model to explore the potential impacts of different approaches especially less disruptive interventions on hospital overcrowding. The expected outcome based on theoretical prediction of this method was evaluated, that is, applying a less disruptive intervention is often enough, and more cost effective, to reduce the risk level of hospital congestion. Making a small number of extra beds available was a superior solution compared to discharging approaches to reduce crowding in hospitals. In addition, the virtual implementation approach enabled testing of the method at a more detailed level, thereby revealed some interesting findings difficult to achieve theoretically, such as discharging smaller numbers of medical inpatients, rather than surgical inpatients, a day earlier when R reaches a threshold, would bring more benefits in terms of congestion reduction for the hospital.

## Supplementary Information

Supplementary Information.

## Data Availability

The patient flow data used for this work were obtained with approval by the Ethics Committee, SA Health Office for the Research Study ‘Congestion recovery and optimisation of patient flows’ (Application number 475.13). These data were used under license for the current study, and so are not publicly available. Data are however available from the authors upon reasonable request if you contact Research Governance & Ethics - Office for Research, Southern Adelaide Local Health Network and get the permission.

## Abbreviations

ED: Emergency Department
HESMAD: Hospital Event Simulation Model: Arrivals to Discharge
SI: Supplementary Information
