# Supplementary material for: A virtual evaluation of options for managing risk of hospital congestion with minimum intervention
Source: Sci Rep. 2022 Aug 27;12:14634. doi: 10.1038/s41598-022-18570-5 (PMC9420155; doi:10.1038/s41598-022-18570-5)
Supplement: Supplementary file 1 — Supplementary Information. [file 41598_2022_18570_MOESM1_ESM.docx]

## A virtual evaluation of options for managing risk of hospital congestion with minimum intervention

^*1^Wanxin Hou, ^2^Shaowen Qin, ^3^Campbell Henry Thompson

*1. School of Information Science and Technology, Research Centre for Intelligent Information Technology, Nantong University, Jiangsu, China.

Email: [houwanxin0403@ntu.edu.cn](mailto:houwanxin0403@ntu.edu.cn)

2. College of Science and Engineering, Flinders University, Adelaide, Australia. Email: Shaowen.Qin@flinders.edu.au

3. School of Medicine, University of Adelaide, Adelaide, Australia. Email: campbell.thompson@adelaide.edu.au



Supplementary Information (SI)

**SI1. Histogram of Nt**

Since *N_t_* is random, the negative binomial distribution and the normal distribution were employed to fit the histogram exhibited in SI-Fig. 1. The normal distribution was selected.


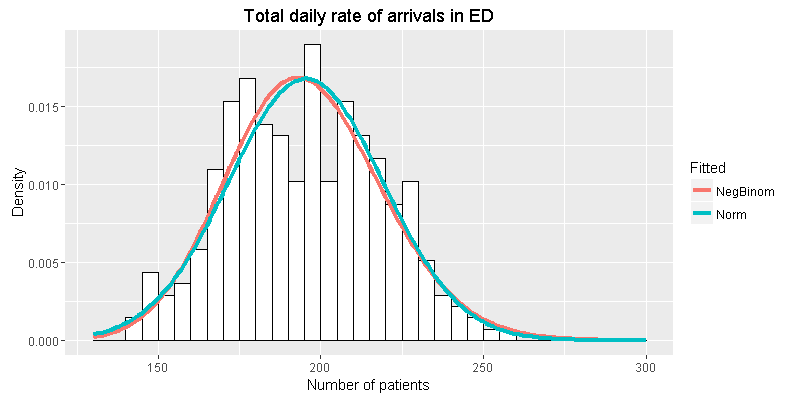


SI-Fig. 1 Histogram of Nt and two matched distributions.

**SI2. An example of R(C) change with different admission and discharge rates**


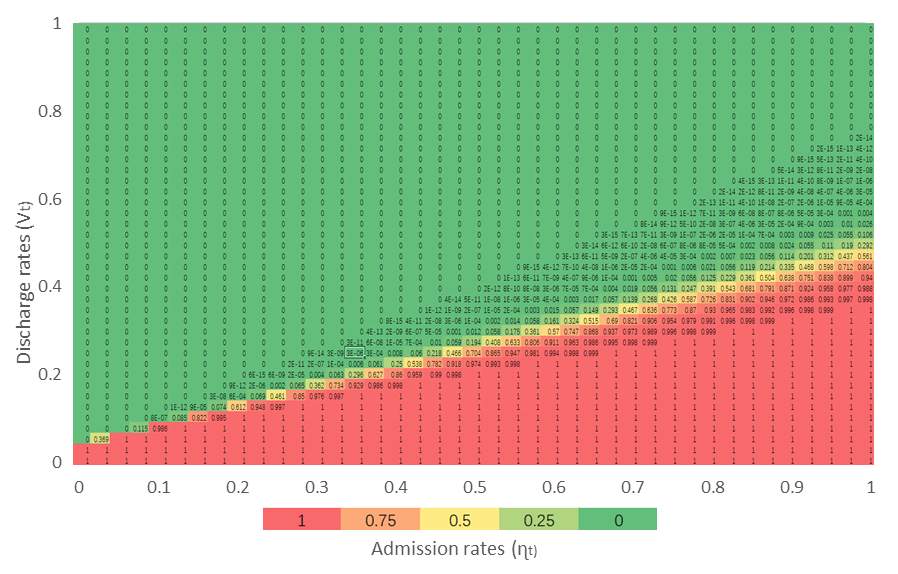


SI-Fig. 2 The R(C) change with different admission and discharge rates

SI-Fig. 2 illustrates the R(C) change of an example with different admission and discharge rates (the midnight occupancy of the previous day M_t-1_ = 595, Elective patients E_t_: 28, C = 600) of a large Australia metropolitan hospital. The horizontal and vertical axes represent admission rates $\eta_{t}\in\left( 0, 1 \right]$ and discharge rates $v_{t}\in(0,1]$ respectively. The green indicates low risk values and the red area indicates high risk values. The thin wedge named “hospital instability wedge” is indicated by the colors changing from red to green. In SI-Fig. 2, it has been found that the thinner the wedge, the more sensitive is the risk R(C) changing from red to green. Theoretically, smaller changes in the numbers of admitted or discharged patients exhibit more effective and sensitive impacts on R(C) which indicates the probability of hospital congestion occurrences because of the relative thinness of the wedge.

**SI3. HESMAD structure**


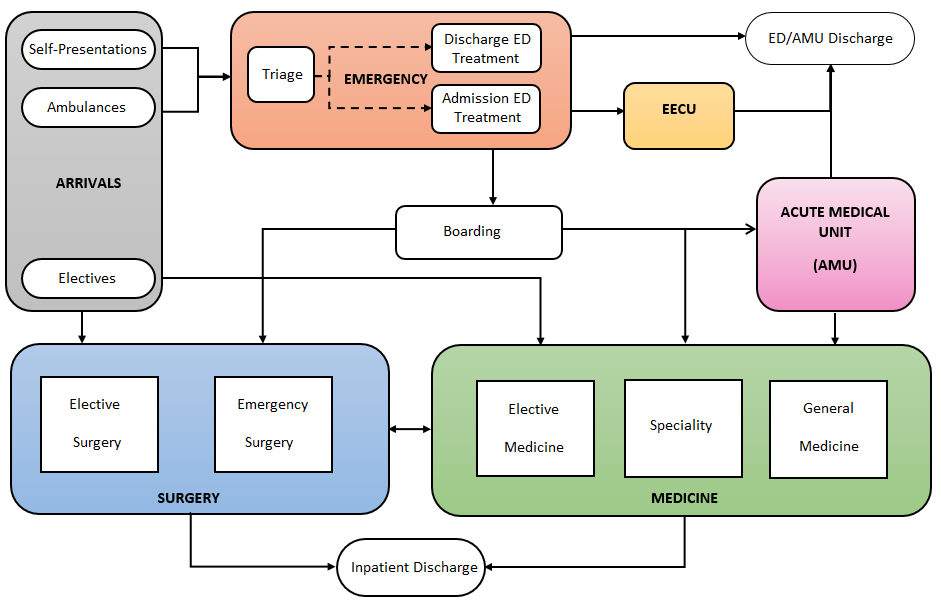


SI-Fig. 3 Overview of the HESMAD structure.

**SI4.The Process and modules of HESMAD**

Different types of patients go through physical units (SI-Table 1) which were represented as modules in the model. All processes, interactions and behaviours between patients and the hospital were included in each module to imitate patient journeys.

The linked modules catch patient pathways when they go through the physical units in the hospital. The process begins when all patients except electives arrive in the ED. A triage score is assigned to indicate the severity of patient condition and to decide treatment stream and queuing priority. Some patients are discharged when they finish ED treatment. Other patients are admitted into the hospital inpatient units. In the inpatient departments, 330 base beds and 8 flex beds were modeled in the simulation model. Patients are placed in a queue when beds are not available in relevant treatment areas. Probability distributions obtained from historical data were used to reflect patient state and their LOS in different areas of the hospital. The final process of discharging patients involves releasing resources. Monitoring different indicators such as the number of beds occupied allows one to evaluate hospital congestion and investigate the potential influence of strategical interventions on hospital overcrowding.

| **Physical Unit Description** |
| --- |
| **1. ED** Emergency department (18 beds for admission stream, 18 beds for discharge stream): models initial care of patients; two arrival modes including self-presentation or by ambulance. |
|  |
| **2. EECU** Extended emergency care unit (10 beds): fast-turnover inpatient ward for patients who require up to 24 hours of monitoring within the ED before being sent home. |
|  |
| **3. AMU** Acute medical unit (30 beds): assessment unit for general medical and acute care elderly patients. |
| **4.Medicine** Largest inpatient division within the hospital (170 beds). Treats patients requiring emergency hospital care for medical conditions with complex and multi system disease. Three treatment streams are modelled based on how patients enter the division: speciality – referred directly from ED, general medicine – patients underwent AMU assessment and, elective – non-emergency arrival undergoing non-surgical elective procedure requiring overnight care. |
|  |
|  |
|  |
| **5. Surgical** Other primary inpatient division (130 beds) that treats patients requiring surgery. Two treatment streams based on mode of entry: emergency surgery and elective surgery. |
|  |

SI-Table 1-1 the description of physical units of the HESMAD

The main state variable we are modelling is occupancy on a given day, τ∈N^0^, denoted by vector **X**(*τ*). Conceptually, the model which computes **X**(*τ*, ℓ, *i*) reported in SI-[Fig. 3](https://www.sciencedirect.com/science/article/pii/S1569190X16302088#fig0001) is the balance equation.

$\mathbf{X}\left( \tau\mathcal{, l,}i \right)\boldsymbol{=}f(\mathbf{X}\left( \tau-1, \mathcal{l}, i \right),\mathbf{A}\left( \tau,m \right),\mathbf{D}\left( \tau\mathcal{, l,}i \right))$ (1)

where **A**(*τ, m*) and **D**(*τ*, ℓ, *i*) are vectors representing the arrivals and discharges for day *τ*, and are realisations of the corresponding stochastic processes that are modelled in their individual process modules. We note that the use of vectors in [(1)](https://www.sciencedirect.com/science/article/pii/S1569190X16302088#eq0001) is indicative of the complex nature of the hospital system where each of the vectors **X, A** and **D** comprises sub-populations representing similar events (e.g., arrivals) but vary sufficiently in their physical and modelled behavior. For occupancy **X** and discharge **D**, ℓ∈{1,…,5} represents a division/area within the hospital where each ℓ corresponds to one of HESMAD’s physical units (see SI-[Table 1](https://www.sciencedirect.com/science/article/pii/S1569190X16302088#tbl0001)-1) and i∈I(ℓ)∈N represents a particular stream within the division ℓ. For arrivals the vector **A**(*τ, m*) comprises three elements *Am*(*τ*) where m={1,2,3} represents different mode of patient arrival and corresponds to ambulance, self-presentation and elective arrivals, respectively.

For simplicity, we use the variable *t* ∈ [0, 24) to represent the hourly time during day *τ*, where τ+t actually denotes τ+t/24. When no ambiguity exists, we drop the *τ* from the argument and simply use the daily time *t*. Finally, Xℓ(t)=∑i∈I(ℓ)X(t,ℓ,i) represents the aggregated occupancy across all streams *i* of physical unit ℓ at time *t*.

Since HESMAD includes many [random variables](https://www.sciencedirect.com/topics/engineering/random-variable-xi) the issue of the use of [parametric](https://www.sciencedirect.com/topics/mathematics/parametric) versus empirical distributions arises naturally. In constructing and validating HESMAD we used both parametric (e.g., arrival process) and empirical distributions (e.g., see SI-[Table 1-2](https://www.sciencedirect.com/science/article/pii/S1569190X16302088#tbl0002), triage, ED streams and treatment process modules). There is a natural temptation to use parametric distributions whenever they provide very good fits to observed trends because these may be easily transferrable to analogous variables in other hospitals. We followed standard statistical measures for “goodness of fit” to make the above determination ^17^.

| **Process Module** | **Description** |
| --- | --- |
| **Arrivals** | Generates emergency and elective patients within the simulation. |
| **Triage** | System for defining the urgency of clinical review and the initiation of definitive treatment. Empirical distributions based on mode of arrival. Patients are assigned a triage upon arrival which ranges from 1-5. A low score indicates requiring immediate attention and a high score corresponds to a less critical case. |
| **ED buffer** | Post-triage patient queue. Order of service is based on assigned triage score, priority given to triage 1 and 2 patients. |
| **ED streams** | Assesses, treats and/or stabilizes emergency patients. Contains two, physically separated, streams based on likelihood of admission for inpatient care. Estimated treatment time is drawn from empirical distributions based on ED stream, triage score and mode of arrival. |
| **Dispatch** | Allocates patients to modelled inpatient streams. Exploits probabilistic distributions influenced by triage score. |
| **Boarding** | Post ED treatment queue for admitted patients waiting for an inpatient bed. Boarding patients occupy emergency treatment space, reducing ED throughput. |
| **Treatment** | Simulates a patient’s period of treatment associated with their current inpatient stream. LOS is drawn from empirical distributions for relevant stream. |
| **Resources** | Finite resource utilized by patients within physical units. Resources includes ED treatment spaces and inpatients beds. Restricts patient flow between physical units based on availability. |
| **Operating hours** | Schedule module which controls inpatient operation hours relevant to various patient streams. |
| **Discharge** | Controls discharge processes for patients exiting FMC from a physical unit. Includes administrative delay relating to processing a discharge as well as a cleaning delay on freeing up a resource. |

SI-Table 1-2 HESMAD process modules

**SI5. Patients Type**

| Patients type | Description |
| --- | --- |
| Medical patients | Patients receive medical treatment from ED, AMU and transferred from other physical units |
| Surgical patients | Patients receive surgical treatment from ED and transferred from other physical units |
|  |  |
| Long stay patients | Inpatients whose Length of stay over 21 days |

SI-Table 2 Patients Type

**SI6. Historical data of inpatients**

|  | Total numbers | Proportion of patients whose LOS<=21 days | Proportion of patients whose LOS>21 days |
| --- | --- | --- | --- |
| Medical Patients | 21773 | 94% | 6% |
| Surgical Patients | 13630 | 97% | 3% |

SI-Table 3 Historical data of inpatients

**SI7. Length of Stay Distribution of Inpatients**


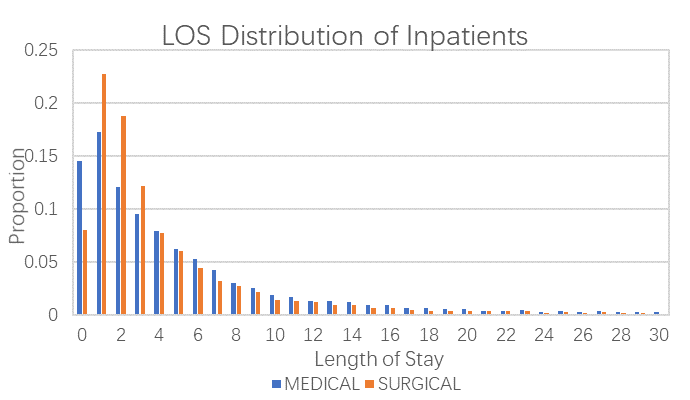


SI-Fig. 4 LOS Distribution of Inpatients

SI-Fig. 4 demonstrated LOS distributions of medical and surgical patients. The horizontal axis shows the LOS of inpatients. The vertical axis presents the proportion of patients with different LOS.

It has been found that there were 21773 medical patients receiving treatment in the medical department for one year which accounted for the total numbers of 46.3% of the total number of patients attending this hospital. Surgical patients numbered 13630, or 29% of the total. There are 17.3% more medical patients than surgical patients. According to historical data, there are about 94% of medical patients and 97% of surgical patients whose LOS are less than 21 days.

**SI8. Executing the scenario with different R(C) value**

SI-Table 4 exhibits the results of executing scenario 11 when R(C)>0.75, 0.85 and 0.95.

| Scenario No. | Midnight Occupancy | Numbers of red days | Numbers of amber days | Numbers of green days | Total Numbers of day that R(C)>0.75, 0.85 and 0.95 | Red day reduction/Discharge |
| --- | --- | --- | --- | --- | --- | --- |
| 11-1  (R>0.75) | 304.89 | 49.43 | 228.76 | 88.81 | 164.2 | 0.19 |
| 11  (R>0.85) | 306.64 | 52.21 | 234.42 | 77.37 | 139.2 | 0.2 |
| 11-2  (R>0.95) | 309.47 | 64.2 | 247.15 | 52.65 | 106.45 | 0.15 |

SI-Table 4 Comparison of discharging 2 medical patients when R(C)>0.75, 0.85 and 0.95.

The total number of days that R(C)>0.75, 0.85 and 0.95 also influences the total numbers of patients discharged during the simulation period. Red day reduction per discharge was calculated as red days reduction compared to base case scenario divided by total numbers of patients discharged. According to the result in SI-Table 4, red day reduction per discharge of scenario 11 is 0.2 which is higher than scenario 11-1 and 11-2. That is to say, selecting R(C) > 0.85 for the condition maximises occupancy benefits for the least disruption to patient care.
